# Proactive personality and its impact on online learning engagement through positive emotions and learning motivation

**DOI:** 10.1038/s41598-024-79776-3

**Published:** 2024-11-15

**Authors:** Pingting Fu, Chengjin Gao, Xueyi Chen, Zihao Zhang, Jufeng Chen, Dong Yang

**Affiliations:** 1 College of Business, Beijing Institute of Technology, Zhuhai, China; 2 College of Education for the Future, Beijing Normal University, Zhuhai, China; 3 School of Software and Big Data, Changzhou College of Information Technology, Changzhou, China; 4 College of Business Administration, Beijing Normal University - Hong Kong Baptist University United International College, Zhuhai, China

**Keywords:** Proactive personality, Online learning, Engagement, Emotions, Learning motivation, Physiology, Psychology

## Abstract

The rapid change in information technology has made online or blended learning a new norm in higher education. However, the problem of low student engagement in online learning is becoming more prominent. This study explores how college students’ proactive personality affects their online learning engagement through the mediating effects of positive emotions and learning motivation. A survey was conducted on college students (*N* = 1049), and the interrelationships among these variables were analyzed using structural equation modeling. The findings unveiled that: proactive personality positively predicts online learning engagement, positive emotions and learning motivation. Positive emotions and learning motivation significantly positively influence online learning engagement. Moreover, Positive emotions also exert a significant positive impact on learning motivation. In addition, the mediation of positive emotions and learning motivation further strengthened a significant positive correlation between proactive personality and online learning engagement. Implications for theory and practice, including the limitations, were discussed accordingly.

**Supplementary Information:**

The online version contains supplementary material available at 10.1038/s41598-024-79776-3.

## Introduction

Due to the rapid progress of information technology, especially the emergence of generative AI technology, higher education has experienced unprecedented changes, and online and blended teaching has become the new normal of education. Massive Open Online Courses (MOOCs) have stimulated interest in online learning among learners at all levels and in all types of education^1^. Most previous studies have found that online learning promotes learner interaction, boosts cognitive processes, and enhances learning performance^2^. However, other studies found that online learners encounter many challenges, such as time-consuming, less rigorous schedules and designs^3^and insufficient face-to-face communication, leading to low course completion rates and student engagement. Therefore, scholars argue that online learning requires students to have high self-regulation skills^4,5^. Extant research has found that students’ proactive personality traits are particularly important in this self-directed learning environment^6^. A proactive personality is viewed as an individual’s stable tendency to change their environment positively. It is a trait disposition that tends to be proactive, goal-oriented, and unconstrained by environmental forces^7,8^. Inspired by recent active learning approaches^9,10^, many scholars have included proactive personality as an important predictor of academic success in traditional and online learning environments^6,11^. According to those reports, students of proactive personality are more willing to actively seek learning resources and participate in learning discussions, thus maintaining a higher level of learning engagement.

Learning engagement refers to the amount of physical and mental energy students put into their academic experience^12^. It was conceptualized as a positive psychological state that includes energy, absorption, and dedication^13,14^. In online learning environments, most often, the behavioral aspect of learning engagement was studied, where they investigate students’ participation in online learning activities^15–19^, (e.g., the number of video lectures watched, contributions made to discussion forums, the number of video interactions, note-taking, and reflective journals submitted). Previous studies have found that online learning engagement contributes to learning satisfaction, reduces dropout^20,21^, and predicts academic performance^22,23^.

Although studies have explored the relationship between proactive personality and learning engagement, for example, studies have found a significant positive correlation between proactive personality and learning engagement^6^, or between proactive personality and learning performance^24^. Students of proactive personality tend to have higher online learning engagement, demonstrate higher enthusiasm for learning, longer learning time, and more learning interactions^11^. Research also confirms that a proactive personality predicts students’ online learning engagement positively. However, the relationship between college students’ proactive personality and online learning engagement has yet to be systematically studied, and most of the studies have focused on traditional face-to-face teaching environments or investigated such relations in the context of work engagement^25–27^.

Among the factors affecting learning engagement, positive emotions and learning motivation are two important aspects. Positive emotions were defined as positive feelings individuals experience when their needs are met^28^. Positive emotions significantly contribute to students’ learning motivation, which, in turn, promotes online learning engagement^29,30^. Motivation was conceptualized as an internal force that drives students to continue learning and is closely related to learning engagement^31,32^. It has been shown that positive emotions and motivation have a significant positive effect on learning engagement, and there is a mutually reinforcing relationship between positive emotions and motivation, i.e., positive emotions enhance motivation, and high motivation leads to more positive emotions^33^. When students experience positive emotions such as pleasure and interest in the learning process, they are more likely to develop intrinsic motivation, which enhances engagement with the content^34^. Although studies have explored the effects of positive emotions and learning motivation on engagement in learning, their possible mediating role between proactive personality and online learning engagement has yet to be fully explored.

The present study aims to fill this research gap by exploring how college students’ proactive personality affects their online learning engagement through positive emotions and study motivation and to test whether there is a mediation of this effect. The study hypothesized a positive relationship between proactive personality and online learning engagement and that the chain mediation effects of positive emotions and learning motivation strengthen this relationship. This mediating effect provides new perspectives and explanations for understanding the relationship between proactive personality and online learning engagement. This study is expected to enrich and expand the current research on the relationship between college students’ proactive personality and online learning engagement in at least three ways. *(1)* offering new perspectives for understanding the psychological mechanism of online learning to provide theoretical support and practical guidance for online education; *(2)* helping educators and students better understand and utilize proactive personality, positive emotions and learning motivation to improve the effectiveness of online learning; and *(3)* providing a scientific basis for design online learning platforms that promote students’ learning engagement and learning performance.

## Theoretical framework

### Proactive personality and online learning engagement

The relationship between proactive personality and online learning engagement is gaining attention in education. Existing studies on traditional learning environments have confirmed the positive relationship between proactive personality and the behavioral and cognitive aspects of learning engagement^11,35^, and some studies have demonstrated a positive link between them in online learning environments^36^. Research has shown that college students with highly proactive personalities show higher engagement in online learning^6^. In addition, the positive correlation between proactive personality and learning engagement has been verified in different learning environments^6,24^. In summary, the following hypothesis is proposed.

**H1: **Proactive personality has a significant positive effect on online learning engagement.

#### The mediating role of positive emotions

According to the broaden-and-build theory of positive emotions^37–42^, positive emotions can activate processes that broaden the range of possible engagements, such as exploring, playing, or thinking activity sequences of ideas and actions, and build personal resilience^43^. Highly proactive personalities are more effective in mobilizing energy and emotions to cope with dilemmas and challenges^44^. In summary, the following hypothesis is proposed.

**H2: **Proactive personality has a significant positive effect on positive emotions.

Besides, the influence of positive emotions on online learning engagement is also supported by research. Studies have shown that self-control and emotions, intrinsic factors in individual college students, can influence learning engagement, impacting academic procrastination and burnout^45^. Conservation of resources theory (COR) views emotions as energy resources for individuals to make resource investments^46^but does not shed light on the mechanisms by which emotions play a role in resource conservation. In contrast, the broaden-and-build theory of positive emotions^38^states that positive emotions can expand an individual’s attention and cognitive scope, motivating the individual to approach and explore unique ways to build enduring personal resources to cope with adversity. For students, positive emotions have the potential to build and facilitate their physical, psychological, and social resources. For instance, high positive emotions in students have been associated with increased focus on learning tasks^47^, greater willingness to invest energy, heightened persistence in learning, and improved psychological well-being, facilitating students’ learning engagement^48–50^. In summary, the following hypothesis is proposed.

**H3: **Positive emotions have a significant positive effect on online learning engagement.

Moreover, positive emotions may mediate the relationship between proactive personality and online learning engagement. A proactive personality facilitates positive emotions, enhancing learning engagement by stimulating positive emotions^30,38^. The extraversion that individuals with high proactive personalities possess may be the mechanism that drives their positive emotional experiences, and positive emotional states, in turn, create a favorable psychological environment for individuals to learn. Based on this, we have the following hypothesis.

**H4: **Positive emotions partially mediate the relationship between proactive personality and online learning engagement.

### The mediating role of learning motivation

Research has found that motivation is a complex umbrella concept influenced by the learner’s internal and external psycho-social factors^51^(presented in the learner’s social and natural environment). According to the framework of the self-determination theory^52^(SDT), the self has a dynamic role in the motivational process, and the individual is free to choose their motivation based on the environment and needs, which are further motivated by a positive environment and constantly satisfied needs. Motivation for learning is attributed to good academic achievement and students’ perceptions of pleasure, joy, and satisfaction, which further encourages their commitment to learning^53^. Individuals with highly proactive personalities are usually more motivated to learn because they pursue personal growth and goal achievement. In summary, the following hypothesis is proposed.

**H5: **Proactive personality has a significant positive effect on online learning motivation.

Extant studies on the impact of learning motivation on online learning engagement have begun to analyze such topics from the perspective of how external motivators of learning motivation are gradually internalized into an individual’s internal motivation process^31,54^. Students with higher learning motivation are likely to have higher levels of learning engagement despite facing learning difficulties and risks^55^. Meanwhile, motivated students are more inclined to set higher goals and expectations, to be motivated to complete learning tasks, and more likely to have higher levels of learning engagement^56,57^. Other researchers suggested that motivation affects students’ classroom engagement, which in turn affects students’ (overall) learning engagement^58,59^, and students with strong motivation to learn are more likely to demonstrate high levels of engagement in online learning environments. In addition, learning motivation mediates the relationship between perceived teacher support and learning engagement^60,61^. Therefore, the following hypothesis is proposed.

**H6: **Learning motivation has a significant positive effect on online learning engagement.

Individuals with proactive personalities may be more engaged in online learning because they have higher motivation to learn§. When students have cultivated a higher proactive personality, and as the influence of proactive personality on individuals increases, so does their learning motivation. A stronger learning motivation enhances students’ interest in online learning, leading to increased online learning engagement^62^. In summary, the following hypothesis is proposed.

**H7: **Learning motivation partially mediates between proactive personality and online learning engagement.

### Chain mediation effect of positive emotions and learning motivation

The relationship between positive emotions and academic motivation has also been the focus of research. Scholars believe that emotions are not only an expression of behavior but also a representation of some adaptive motivational factors^38,63^. Emotions influence college students’ learning motivation^64^. For example, positive emotions stimulate and sustain learners’ intrinsic motivation, which promotes (positive) learning behavior^65^. When in a positive emotional state, students become willing to learn, good at learning, and develop a strong interest in learning^66^. Therefore, positive emotions can play a positive role and further impact learning motivation. Accordingly, the following hypothesis is proposed.

**H8: **Positive emotions have a significant positive effect on learning motivation.

According to the SDT, a proactive personality increases students’ learning motivation^53^. Learning motivation becomes stronger as emotional positivity increases. This study examines how positive emotions and learning motivation work together as chain mediators to influence the relationship between proactive personality and online learning engagement. That is, a proactive personality ultimately influences online learning engagement through the interaction of positive emotions and learning motivation. Accordingly, the following hypothesis is proposed.

**H9: **There is a chain-mediation effect of positive emotion and learning motivation between proactive personality and online learning engagement.

**Fig. 1:**
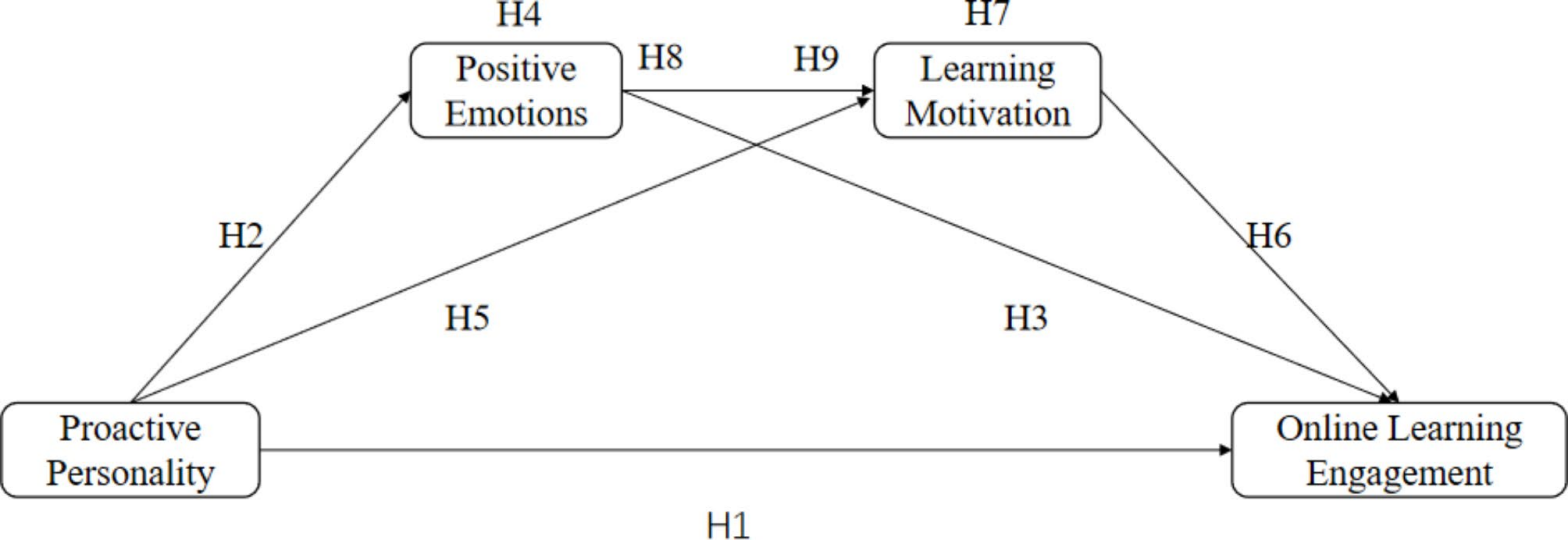
The proposed research model.

Details regarding all the hypotheses and research models are shown in Fig. 1.

## Methodology

### Questionnaires/tools

We used existing scales to collect data. For this study, several modifications were made to the scales. Since the original instruments were in English while our participants were Chinese speakers, we employed back-translation to guarantee the survey questions’ validity and appropriateness. The details are as follows:

### Proactive personality

The scale was based on the Proactive Personality Scale developed by Bateman and Crant^67^, which has been subjected to numerous empirical tests and has high reliability and validity^68^. The scale was scored on a 5-point Likert scale (1 = strongly disagree; 5 = strongly agree), and ten items were selected for the scale (e.g., *I am constantly looking for new ways to improve my life*). The Cronbach coefficient for this scale in this study was 0.948.

### Learning engagement

The scale was selected from the short work engagement scale developed by Schaufeli et al^69^., which consists of 9 questions. An example of the question was, “*At my work*,* I feel bursting with energy.*” The scale has three dimensions: vigor, dedication and concentration, with internal consistency coefficients of 0.79, 0.89 and 0.72, respectively. The scale features a 5-point Likert scale, and the higher the score, the better the college students’ online learning engagement. The Cronbach coefficient of the scale in this study was 0.948.

### Positive emotions

The Positive and Negative Affect Schedule (PANAS) proposed by Watson et al^70^. was adopted to measure positive emotions. The scale consisted of two dimensions (i.e., positive and negative affect), each containing ten emotion descriptors. Since the present study only concerns positive emotions, the ten items of the positive affect dimension of the PANAS were chosen (e.g., *Interested*,* excited*,* inspired*). Similarly, questions were asked on a 5-point Likert scale (1 = very little or not at all; 5 = very much). The Cronbach coefficient in this study was excellent at 0.937.

### Learning motivation

Learning motivation was measured using the Online Learning Enrollment Intentions (OLEI) Scale developed by Kizilcec and Schneider^71^. The scale aimed at collecting feedback from learners online from aspects such as social needs, skill enhancement, or interest exploration. Overall, there are 13 items. For this study, three items (i.e., *relevant to the job*,* for a career change*,* and to meet new people*) were deleted since they are not the concern in this study. Therefore, ten items were retained in the final scale. The scale was scored on a 5-point Likert scale. The Cronbach’s coefficient was good at 0.897.

### Sample and procedure

A total of 1049 participants (67.3% female) were included in this study, comprising college students across various academic years. The sample encompassed first-year to graduate students, with the highest representation from sophomores (35.4%), followed by first-year students (34.3%), juniors (18.7%), seniors (8.8%), and graduate-level students (2.9%). Regarding academic majors, arts and sports constituted the largest subgroup (28.5%), followed by science and engineering (25.5%), literature and history (19.4%), and economics and management (10.1%). The distribution of the number of courses taken on the online learning platform during the observation period indicated that 1–4 courses accounted for 54.4% of participants, 13 or more courses for 8.3%, 5–8 courses for 27.2%, and 9–12 courses for 10.1%.

As stated above, the questionnaire was developed using a combination of multiple scales. The survey was sent using the online data collection platform Wenjuanxing (https://www.wjx.cn/). Participants filled out the questionnaire using mobile devices. All subjects participated voluntarily and were informed of the study’s purpose and they can stop participating whenever they feel uncomfortable. Informed consent was obtained from all subjects following the Declaration of Helsinki. The research protocol of this study was approved by the Humanities and Social Sciences Ethics Review Committee of the first author’s university.

### Data analysis

Descriptive statistics, ANOVA, correlation, regression, and mediation effect tests were conducted using SPSS 25.0 software and its official plug-in PROCESS 4.0. Gender, grade level, major category, and number of courses taken were chosen as the control variables, and the relationship between the four components of proactive personality, online learning engagement, positive emotions, and learning motivation was explored systematically.

### Results

#### Descriptive statistics

The descriptive analysis results show significant gender differences in proactive personality, online learning engagement and positive emotions. The means of male students are higher than those of female students, indicating that male students have a higher proactive personality, more positive emotions, and a higher degree of online learning engagement. Still, male and female students are similar in motivation for learning. In addition, there were significant differences in the levels of proactive personality, online learning engagement, positive emotions, and motivation across grades. First-year college students have higher means for online learning engagement, proactive personality, positive emotions, and motivation than their peers. Significant differences also exist among different major categories. Students majoring in science and engineering have higher levels of proactive personality and positive emotions. Moreover, they tend to be more motivated to learn and ultimately commit to online learning. Last, there were no significant differences in the proactive personality, online learning engagement, positive emotions, and motivation of the subjects for different numbers of courses taken during the use of the online platform (see Table 1).

**Table 1:** Descriptive statistics (*N* = 1049).

		Online learning engagement M(SD)	Motivation M(SD)	Proactive personality M(SD)	Positive emotions M(SD)
Gender	Male	3.11(0.87)	3.97(0.69)	3.81(0.84)	3.71(0.78)
	Female	2.90(0.82)	3.91(0.59)	3.54(0.84).	3.51(0.71)
	T-test	3.90***	1.54	4.94***	4.29***
Grade	First-year	3.15(0.84)	4.07(0.60)	3.77(0.86)	3.72(0.77)
	Second-year	2.92(0.84)	3.92(0.61)	3.60(0.86)	3.5(0.74)
	Third-year	2.85(0.82)	3.81(0.60)	3.51(0.80)	3.5(0.66)
	Fourth-year	2.66(0.76)	3.67(0.61)	3.41(0.82)	3.3(0.70)
	Graduates	3.09(0.91)	3.93(0.83)	3.72(0.83)	3.57(0.71)
	F-test	10.56***	9.22***	5.39***	6.51***
Major	STEM	2.93(0.82)	3.90(0.60)	3.65(0.78)	3.6(0.70)
	Arts & sports	3.11(0.83)	4.02(0.65)	3.81(0.79)	3.72(0.71)
	Literature & history	2.87(0.83)	3.86(0.61)	3.47(0.86)	3.4(0.72)
	Economics & management	2.87(0.83)	3.89(0.57)	3.59(0.86)	3.52(0.71)
	Others	2.94(0.90)	3.93(0.64)	3.50(0.97)	3.4(0.84)
	F-test	2.59*	3.31*	6.57***	5.48***
Course Taken	1–4	2.99(0.84)	3.95(0.60)	3.63(0.84)	3.57(0.74)
	5–8	2.94(0.81)	3.93(0.62)	3.64(0.87)	3.6(0.73)
	9–12	2.95(0.84)	3.91(0.66)	3.63(0.87)	3.5(0.70)
	13 or more	2.90(0.96)	3.85(0.71)	3.57(0.84)	3.52(0.81)
	F-test	0.78	0.48	0.14	0.33

Correlation analysis was carried out to confirm the hypothesis of the relationship between the variables. As shown in Table 2, the results indicate a positive correlation between active personality and positive emotions, stronger motivation, and online learning engagement. Moreover, positive emotions are significantly and positively correlated with motivation and online learning engagement, and motivation is significantly and positively correlated with online learning engagement. Meanwhile, gender and grade negatively correlate with active personality, positive emotion, stronger motivation, and online learning investment. Lastly, the major category correlates negatively with active personality and positive emotion. Unfortunately, the number of courses taken online does not correlate significantly with the variables. Based on the results, it is necessary to control the variables of gender, grade, and major category when conducting regression analysis. Refer to Table 2 for details.

**Table 2:** Correlation of study variables (*N* = 1049).

Variables	1	2	3	4	5	6	7	8
Gender	1.000							
Grade	−0.002	1.000						
Major	0.242^**^	− 0.065^*^	1.000					
Course	− 0.004	0.259^**^	0.067^*^	1.000				
LM	− 0.068^*^	− 0.190^**^	− 0.005	− 0.030	1.000			
OLE	− 0.123^**^	− 0.157^**^	− 0.035	− 0.025	0.684^**^	1.000		
AP	− 0.153^**^	− 0.131^**^	− 0.085^**^	− 0.007	0.623^**^	0.759^**^	1.000	
PE	− 0.145^**^	− 0.115^**^	− 0.106^**^	0.011	0.558^**^	0.677^**^	0.759^**^	1.000

*Notes*: LM = Learning motivation; OLE = Online learning engagement; AP = active personality; PE = positive emotions.

### Relation between proactive personality and online learning engagement

Active personality significantly and positively predicted online learning input (β = 0.413, *p* < 0.001), and **H1** was confirmed. This was demonstrated by regression analysis with online learning input as the dependent variable and positive emotion, learning motivation, and active personality as the independent variables.

**Table 3:** Regression analysis of the variables in the chaine mediation model.

Outcome variable	Predictor variable	*R* ^2^	ΔR^2^	F	β	SE	Sig
PE	AP	0.625	0.586	1629.325	0.682	0.017	0.000
LM	AP	0.423	0.393	357.334	0.322	0.028	0.000
	PE				0.198	0.032	0.000
OLE	AP	0.695	0.659	749.769	0.413	0.029	0.000
	PE				0.224	0.032	0.000
	LM				0.437	0.031	0.000

LM = Learning motivation; OLE = Online learning engagement; AP = active personality; PE = positive emotions.

### Mediating effects of positive emotions and motivation on relations between proactive personality and engagement

A series of regression analyses were conducted to test the hypothesis as proposed above. First of all, when putting positive emotions as the dependent variable and proactive personality as the independent variable, the results (as shown in Table 3) indicated that proactive personality significantly and positively predicted positive emotions (β = 0.682, *p* < 0.001); thus, **H2** was validated. Then, with motivation as the dependent variable and proactive personality and positive emotions as the independent variables, we found that proactive personality significantly positively predicted learning motivation (β = 0.322, *p* < 0.001), and **H5** was verified. Positive emotions significantly predicted motivation (β = 0.198, *p* < 0.001), and **H3** was verified. Moreover, learning motivation substantially and positively predicted online learning engagement (β = 0.437, *p* < 0.001), **H6** was testified. Lastly, **H8** was verified since results showed that positive emotions substantially predicted online learning engagement (β = 0. 224, *p* < 0.001). Gender, grade level, major, and number of courses taken were used in the regression analysis as a control variable.

This study found significant correlations between proactive personality, positive emotions, motivation, and online learning engagement, aligning with the selection of mediating variables. The mediating effects were examined using the SPSS plug-in PROCESS 4.0 by Hayes^72^. Model 6 was chosen for a Bootstrap method with 5000 tests applied to a sample size of 1049 and a confidence interval set at 95% for chain mediation effect analysis. The results (refer to Table 4) indicated a significant direct effect (β = 0.414, 95% CI = [0.356, 0.472]) and indirect effect (β = 0.357, 95% CI = [0.304, 0.412]), influencing online learning engagement through three paths: Path 1, proactive personality → positive emotions → online learning engagement (β = 0.153, 95% CI = [0.106, 0.205]), supporting **Hypothesis 4;** Path 2, Proactive Personality → Motivation to Learn → Online Learning Engagement (β = 0.142, 95% CI = [0.107, 0.180]), validating **Hypothesis 7**; and Path 3, Proactive Personality → Positive Emotion → Motivation to Learn → Online Learning Engagement (β = 0.061, 95% CI = [0.040, 0.085]), confirming **Hypothesis 9**. In addition, a table summarizing all the hypotheses tested in this study was provided in Table 5.

**Table 4:** Mediation effect test between proactive personality and online learning engagement.

	Effect	SE	BootLLCI	BootULCI
Direct effect	0.414	0.029	0.356	0.472
Total indirect effect	0.357	0.027	0.304	0.412
Pathway 1 (PP - PE - OLE)	0.153	0.025	0.106	0.205
Path 2 (PP- LM- OLE)	0.142	0.019	0.107	0.180
Pathway 3 (PP - PE - LM- OLE)	0.061	0.012	0.040	0.085

PP = proactive personality; OLE = Online learning engagement; LM = Learning motivation; AP = active personality; PE = positive emotions.

**Table 5:** Summary of hypothesis testing.

Hypothesis	Supported	Effect size	Finding
H_1_	Yes***	Large	Proactive personality has a significant positive impact on online learning engagement.
H_2_	Yes***	Large	Proactive personality has a significant positive impact on positive emotions.
H_3_	Yes***	Medium	Positive emotions have a significant positive impact on online learning engagement.
H_4_	Yes***	Medium	There is a significant positive correlation between proactive personality and online learning engagement, which is strengthened through the mediating role of positive emotions.
H_5_	Yes***	Medium	Proactive personality has a significant positive impact on learning motivation.
H_6_	Yes***	Large	Learning motivation has a significant positive impact on online learning engagement.
H_7_	Yes***	Medium	There is a significant positive correlation between proactive personality and online learning engagement, which is strengthened through the mediating role of learning motivation.
H_8_	Yes***	Medium	Positive emotions have a significant positive impact on learning motivation.
H_9_	Yes***	Small	There is a significant positive correlation between proactive personality and online learning engagement, which is strengthened through the serial mediating role of positive emotions and learning motivation.

### Discussion

This study investigated the complex interplay between proactive personality, positive emotions, motivations, and online learning engagement.

A proactive personality significantly and positively predicts positive emotions, learning motivation, and, eventually, online learning engagement. The chain mediation effect of positive emotions and learning motivation further strengthened Morover, relations between proactive personality and online learning engagement. Our results have implications for both theory and teaching practice.

### Implications for theory

The study reveals that college students with a proactive personality significantly and positively influence their online learning engagement, aligning with earlier research emphasizing the pivotal role of proactive personality in education^24,35,67^. Highly proactive students manifest their increased autonomy through active engagement in learning, exemplified by proactive information gathering^73^, knowledge sharing during the learning process, and enhanced goal adherence^74^. The results also demonstrated a significant correlation between proactive personality, positive emotions, and online learning engagement. According to COR theory, positive emotions can be described as an energy resource influenced by a proactive personality^75^. For example, individuals with highly proactive behaviors are predisposed to experiencing satisfying and enjoyable emotions and are more inclined to adopt positive strategies to cope with challenges, resulting in enhanced positive emotions. Positive academic emotions support students’ cognitive development, sustain their attention, and boost enthusiasm for studying^47,76,77^.

Our study also found positive emotions mediate between proactive personality and online learning engagement; higher levels of proactive personality and positive emotions correspond to higher levels of online learning engagement. These findings validate Fredrickson’s broaden-and-build theory of positive emotions^38^, proposing that positive emotions expand individuals’ attention and cognitive capabilities, motivating them to explore and develop personal resources. In addition, the study reveals that students with proactive personalities are more likely to experience positive emotions, enhancing their personal resources (e.g., motivation and engagement in online learning). Furthermore, the research confirms the positive relationship between motivation and online learning engagement, consistent with prior studies^78,79^. Learning motivation encompasses the motives driving students to pursue specific activities or goals^80^. Scholars have examined the impact of motivation on learning engagement, indicating that higher motivation corresponds to greater learning engagement^81^. Learning motivation also plays a significant role in proactive personality and online learning engagement, affirming the Self-Determination Theory^52^, which highlights intrinsic motivation as a key driver for individuals to persist in learning. Students with proactive personalities typically exhibit higher motivation for learning and increased online learning engagement. Moreover, proactive personality indirectly impacts online learning engagement through the mediating role of learning motivation.

Proactive personality influences online learning engagement by promoting positive emotions and learning motivation. Importantly, the study uncovers the chain mediation effect of positive emotions and learning motivation between proactive personality and online learning engagement. This suggests that a proactive personality contributes to online learning engagement directly and indirectly (by stimulating positive emotions and enhancing learning motivation). For instance, positive emotions enhance students’ overall well-being, fostering greater enthusiasm and creativity for learning and enhancing their ability to overcome setbacks and challenges in learning^82^. This finding offers new insights into how proactive personality influences online learning engagement and underscores the importance of nurturing students’ proactive personalities in online learning environments.

### Implications for teaching practice

The study results show significant differences in grade, gender and professional categories, which deserve to attract the attention of the school and all walks of life. We have the following suggestions for the deficiencies in the online learning investment of college students, combined with the study’s conclusions and the relevant theory.

First, we found significant differences in proactive personality, learning motivation, positive emotions, and engagement across genders, grades and majors. Specifically, females were lower than males in all indicators. Although scholars have found that the gender gap was diminishing globally^83^, for instance, Eagly and colleague^83^ did a meta-analysis on the changes in gender roles and opinions across seven decades and found a considerable change in gender relations, especially in women’s roles. Their study revealed clear increases in the ascription of communion to women relative to men but a lack of change in agency. Moreover, women also gained in competence relative to men, belief in competence equality has increased over time. In online learning environment, Increasing the Level of engagement to online Learning among female university students. We should enhance their self-efficacy, and increase their self-efficacy through online exercise and self-reflection; In addition, we should enhance their learning motivation, positive emotions, and self-reflection, to strengthen learning motivation by guiding female students to find their intrinsic interests and needs and to promote learning engagement through self-motivation and rewards.

Regarding grades, we found a decreasing momentum in proactive personality, learning motivation, positive emotions, and engagement during college years. Such gaps were also identified among students of various majors: arts & sports were relatively higher in aspects researched, while others such as STEM and Business & management were significantly lower in both learning motivation, positive emotions and engagement. This is not surprising since recent study on college students’ competences also evidenced that Chinese STEM majors (e.g., computer science and electrical engineering) university students (among those of Russian and India) do not gain critical thinking skills over four years^84^. These gaps in skill levels and gains provide insights into the global competitiveness of STEM university students across nations and institutional types. Moreover, in China, this could be explained since first-year students enter the university campus, maintain a high sense of freshness, and actively explore the learning and lifestyle to adapt to the university (Author, Year). Moreover, the school pays more attention to the entrance education of new students and is more strict in shaping the daily behaviors and habits of new students, classroom management, etc.

such engagement patterns were also identified in previous studies (e.g., Author, Year; Author2, Year). (When online learning differences among majors, find some references). Therefore, further studies are needed to identify specific difficulties encountered in online learning across all grades and majors.

Finally, our study suggests the importance of enhancing positive emotions online learning motivation of college students. It is found that high levels of proactive personality benefit college students’ engagement in online learning, individuals who cultivate proactivity in themselves, both in their studies and lives, will be motivated to engage in online learning. College students with high proactive personality show higher engagement in online learning^6^. A proactive personality is viewed as an individual’s stabilizing tendency to change the environment positively, and this trait is particularly important in online learning environments because it promotes students’ self-driven and self-management skills. Meanwhile, positive emotions can positively affect college students’ behavior, and those with positive emotions usually increase their interest in learning and, thus, their engagement in online learning. Positive high-arousal moods increase learners’ effort and commitment to learning^85^, allowing learners to experience a good sense of mastery, which leads to an increased willingness to construct knowledge and an increased commitment to learning actively; in contrast, persistent negative moods disengage learners from the learning task^45^. In addition, a proactive personality influences online learning engagement through positive emotions and learning motivation, suggesting that when individuals develop a good proactive personality, they have more positive emotions to enhance their motivation to learn online, which enhances the learning outcomes of online learning. Therefore, it is not enough to rely on a proactive personality to improve college students’ engagement in online learning; it is also necessary for individuals to learn how to mobilize positive emotions and make their motivation clearer to be more efficient.

### Limitations and further directions

Limitations exist in almost all studies, and this study is not an exception. First of all, the majority of participants in this study were first-year students, and the scope of the research object is limited to a certain province in China. Readers need to be cautious when interpreting the findings, and whether the conclusions apply to the general student group requires further investigation. Second, this study only explored the internal mechanism of the chain mediation effect of positive emotion and learning motivation on the relationship between proactive personality and online learning engagement. Other moderating variables, such as preventive orientation and teacher autonomy support, can be introduced in subsequent studies to explore further the relationship between proactive personality and online learning engagement. Last but not least, this study is a cross-sectional study without a follow-up study; future studies are suggested to apply a longitudinal follow-up study design to explore the dynamic long-term effects of proactive personality, positive emotions, and learning motivation on online learning engagement.

### Conclusion

The rapid change in information technology has made online or blended learning a new norm in higher education. Using structural equation modeling, This study investigated the relations between college students’ proactive personality, positive emotions, learning motivation, and online learning engagement. Positive associations between proactive personality and online learning engagement were validated, and moreover, the mediating effort of positive emotion and motivation enforced such relations. Taken together, our results imply the importance of developing positive emotions and learning motivations among college students to foster online learning engagement.

## Electronic supplementary material

Below is the link to the electronic supplementary material.

Supplementary Material 1

## Data Availability

The datasets generated and/or analysed during the current study are available in the Figshare repository, https://doi.org/10.6084/m9.figshare.27092545.
