# Supplementary material for: Proactive personality and its impact on online learning engagement through positive emotions and learning motivation
Source: Sci Rep. 2024 Nov 15;14:28144. doi: 10.1038/s41598-024-79776-3 (PMC11568340; doi:10.1038/s41598-024-79776-3)
Supplement: Supplementary file 1 — Supplementary Material 1 [file 41598_2024_79776_MOESM1_ESM.docx]

**List of abbreviations**

AP - Proactive Personality

COR - Conservation of Resources Theory

LM - Learning Motivation

MOOCs - Massive Open Online Courses

OLE - Online Learning Engagement

PE - Positive Emotions

SDT - Self-Determination Theory
